# Association of the oxidative balance score with obesity and body composition among young and middle-aged adults

**DOI:** 10.3389/fnut.2024.1373709

**Published:** 2024-04-30

**Authors:** Zhiyong Zhu, Hao Bai, Zhaoping Li, Miaomiao Fan, Gang Li, Liyong Chen

**Affiliations:** ^1^Department of Surgery, Shandong Rehabilitation Hospital, Jinan, China; ^2^Department of Nutrition, Qilu Hospital of Shandong University, Jinan, China; ^3^Department of Clinical Nutrition, Shandong Provincial Hospital Affiliated to Shandong First Medical University, Jinan, China; ^4^Department of Health, Shandong University of Traditional Chinese Medicine, Jinan, China; ^5^Department of Vascular Surgery, Shandong Provincial Hospital Affiliated to Shandong First Medical University, Jinan, China; ^6^Department of Toxicology and Nutrition, School of Public Health, Cheeloo College of Medicine, Shandong University, Jinan, China

**Keywords:** oxidative balance, obesity, body composition, fat mass, lean mass

## Abstract

**Objective:**

The oxidative balance score (OBS) is important for determining the cause of obesity and its complications. We aimed to evaluate the association between OBS and obesity and other segmental body composition parameters among young and middle-aged U.S. adults.

**Methods:**

9,998 participants from the National Health and Nutrition Examination Survey 2011–2018 were included. Lean mass percentage (LM%) and FM% were evaluated by dual-energy x-ray absorptiometry. Obesity was defined as body FM% ≥25% in men and ≥ 35% in women. The OBS was scored by 5 pro-oxidant and 21 antioxidant factors. Associations of quartiles of OBS with obesity risk were estimated using multivariable logistic regression models. Multivariable linear regression was conducted to estimate the association between OBS and segmental body composition measures including the arm LM%, leg LM%, torso LM%, whole LM%, arm FM%, leg FM%, torso FM% and total FM%.

**Results:**

Compared to participants in the lowest quartile of OBS, those in the highest quartile of OBS were associated with a lower risk of BMI-defined obesity BMI-defined obesity [0.43 (0.36, 0.50)] and FM%-related obesity [0.43 (0.35, 0.52)]. Additionally, OBS was negatively associated with FM% of the limb and torso but positively associated with the percentage of lean mass (LM%) of the limb and trunk.

**Conclusion:**

OBS was negatively associated with the risk of obesity and segmental FM%, but was positively associated with segmental LM% among US adults, indicating that adhering to an anti-oxidative diet and lifestyle management may be beneficial for preventing segmental obesity.

## 1 Introduction

Obesity, defined as the physically excessive accumulation of body fat (1), has become a major health problem worldwide. The World Health Organization (WHO) standardized body mass index (BMI) over 30 kg/m^2^ in Caucasians as an index of obesity irrespective of age and sex (2). In adult life, however, body composition slowly changes with age, including the degeneration of lean mass and the accumulation of fat tissue (3, 4). Notably, alterations in body composition sometimes may not be accompanied by significant changes in BMI (5). In this context, using BMI alone cannot discriminate individuals with normal weight obesity (NWO), also called occult obesity, from individuals without obesity. A growing number of studies have linked NWO with increased risk of metabolic syndrome (MetS), cardiac morbidity and mortality (6, 7). Therefore, accurate determination of real obesity is vital.

Segmental body composition may help clinicians better distinguishing lean mass from adipose tissue in individual regions (8) and help to define the clinical status of individuals with obesity rather than BMI (9), which has been of interest to clinicians in recent years. Empirical data suggest that lean mass percentage (LM%) is negatively associated with the risk of hypertension (10), diabetes (11), and hypercholesterolemia (12). Given this, when managing our figure, not only should we focus on body weight and BMI but also body composition in different segments.

In addition to a state of positive energy balance, oxidative stress is another vital factor contributing to adipogenesis and lipogenesis in the development of obesity (13, 14). Oxidative stress is a complex process results from the imbalance between protective substances produced by antioxidants and reactive oxygen species produced by pro-oxidants (15), and it is closely related to human diseases, including cardiovascular disease, cancer, and aging (16–18). Due to its significant impact on overall health, evaluating the oxidative balance in individuals is crucial now and in the future. By monitoring oxidative stress status, healthcare professionals can take proactive steps, through a combination of healthy lifestyle and dietary choices, to maintain individuals’ health and well-being, ultimately promoting longevity, improving quality of life.

Lifestyle and dietary intervention exert important effects on the cellular redox status. Mounting evidence has shown that drinking (19), smoking (20) and excessive iron (21) accelerate oxidative stress, while higher consumption of certain nutrients, such as vitamin C (22), vitamin D (23), vitamin E (24), selenium (25), zinc (26), calcium (27) and magnesium (28) protects against oxidative stress related cellular damage. Nevertheless, it is hard to truly reflect the body’s oxidative levels. The oxidative balance score (OBS) was developed for quantifying the physical oxidative stress burden of dietary and lifestyle pro-oxidants and antioxidant factors (16). In general, a higher OBS indicates that antioxidant factors are more dominant than pro-oxidants (29), and OBS was shown to be associated with oxidative stress (30, 31) in previous National Health and Nutrition Examination Survey (NHANES) studies.

However, based on prior studies, there is still on consistent conclusions on the association between OBS and the risk obesity (32, 33). Zahra Noruzi et al’s study showed no significant relationship between OBS and MetS in Iranian (32), while another study revealed that participants in the highest quartile of OBS were less likely to be at risk for MetS than those in the lowest quartile (33). In addition, Yeo et al. (34) reported that Korean individuals with higher OBS had a significantly smaller neck circumference (NC), which was associated with central obesity in the general population (35). Wang et al. (36) reported that higher OBS was significantly correlated with lower risks of abdominal obesity and visceral fat accumulation. Nevertheless, studies about segmental fat mass and lean mass percentage were relatively rare. Notably, single parameters such as waist circumference (WC) and NC cannot reflect specific segmental or local obesity statuses. To further clarify the association between OBS and segmental obesity, we further evaluated fat mass and lean mass percentage in individual regions.

The aim of the present study was to evaluate the relationship between OBS and obesity as well as other segmental body composition parameters using data from the NHANES. We hypothesize that there may be a potential negative relationship between OBS and the risk of obesity and FM%, while positive with LM%.

## 2 Materials and methods

### 2.1 Study population

The subjects of this cross-sectional study were from the NHANES 2011–2018 database. The NHANES is designed to assess the nutritional and health status of the noninstitutionalized population in U.S., and is conducted every 2 years. In every cycle, approximately 5,000 people were selected by a complex multistage sampling strategy, and all participants completed structured questionnaires at home and underwent physical examination at a mobile examination center (MEC). All procedures were approved by the National Center for Health Statistics, and an informed consent form was signed by each subject. All participants signed a written informed consent form.

### 2.2 Oxidative balance score calculation

Both dietary and lifestyle antioxidant/prooxidant factors contributed to OBS (37). The dietary intake information was obtained via 24-h dietary recall interviews at the mobile examination center (MEC). The dietary intake data are used to estimate the types and amounts of foods and beverages consumed during the 24-h period prior to the interview, and to estimate intakes of energy, nutrients, and other food components. In this study, the lifestyle factors associated with OBS included alcohol intake, smoking status, BMI, and physical activity. Participants were categorized according to sex-specific tertiles of energy-adjusted dietary nutrients. Alcohol intake was obtained from the 24-h dietary recall interviews. Nondrinkers, nonheavy drinkers (0 to 30 g/d for males and 0 to 15 g/d for females), and heavy drinkers (≥30 g/d for males and ≥ 15 g/d for females) received 2, 1, or 0 points, respectively. To simultaneously assess passive smoking, the serum concentration of cotinine was used to assess smoking status. BMI was calculated as weight (kg)/height squared (m^2^). Participants were categorized into inactive group (no leisure-time physical activity), insufficiently active group (leisure-time moderate activity 1–5 times per week or leisure-time vigorous activity 1–3 times per week) and active group (those who had more leisure-time moderate or vigorous activity than above) as previously reported (38) and these three groups received 0, 1, and 2 points, respectively.

The pro-oxidants consisted of total fat and iron intake, alcohol consumption, and BMI. The antioxidants included dietary fiber, α-carotene, β-carotene, β-cryptoxanthin, lycopene, lutein+zeaxanthin, riboflavin, niacin, vitamin B6, total folate, vitamin B12, vitamin C, vitamin E, calcium, magnesium, zinc, copper, selenium, vitamin D and physical activity. Finally, the 5 priori defined pro-oxidant and 20 antioxidant factors were equally weighted to construct the OBS. Except for physical activity and alcohol intake, other antioxidants were assigned 0, 1 or 2 points for tertile 1, tertile 2 or tertile 3, respectively, whereas pro-oxidants were reverse scored (Supplementary Table S1).

### 2.3 Outcome

DXA scans were performed on a Hologic QDR 4500 fan beam densitometer (Hologic, Inc., Bedford, MA) according to the manufacture’s guidelines. DXA was administered by trained and certified radiology technologists, and an expert review was conducted. Available data were used to calculate the percentage of total and regional fat mass and lean mass. The LM% was equal to the lean mass (the sum of nonbone and nonfat mass) of the left arm divided by the total weight of the left arm. The FM% was calculated as the fat mass of the left arm divided by the total weight of the left arm. The LM% and FM% of the other segments were calculated with reference to the left arm. According to our preliminary analysis, the limb LM% and FM% on the left and right went hand in hand (Supplementary Figure S1), so the average of LM% and FM% of the limbs were calculated. We focused on the arm LM%, arm FM%, leg LM%, leg FM%, trunk LM%, trunk FM%, total LM% and total FM%. Based on the guideline of the American Association of Clinical Endocrinologists and the American College of Endocrinology, an FM% ≥ 25% for men or an FM% ≥ 35% for women was defined as obesity (39–42). BMI (in kg/m^2^) ≥30 was defined as obesity, calculated as weight (kg) divided by height (m)^2^ (43).

### 2.4 Covariates

Demographics information, including age, sex, race, education, and the ratio of family income to poverty (PIR) was collected. The race was classified as non-Hispanic White, non-Hispanic Black, Mexican American and other. Education level was classified as less than high school, high school or equivalent and college or above. PIR was calculated by dividing family (or individual) income by the poverty guidelines specific to the survey year, and was classified into three categories: <1.3, 1.3–3.5, and > 3.5 based on previous guidelines (44). Self-reported diseases, including hypertension and diabetes, were also included as covariates. The missing data for PIR, hypertension, and diabetes were coded as “unknown”.

### 2.5 Statistical analysis

Continuous variables are presented as the mean with standard error, and categorical variables are presented as weighted percentages. Differences in OBS quartiles were compared by using the weighted chi-square test. Weighted variance analysis was used for continuous variables. Weighted multivariate logistic regression was performed to estimate the independent relationship between OBS and the risk of FM%-defined obesity. Weighted multivariable linear regression analysis was conducted to investigate the associations between OBS and segmental LM% and FM%. Odds ratios (ORs) or β estimates with 95% confidence intervals (95% CIs) were calculated. In Model 1, no covariates were adjusted. Model 2 was adjusted for age (continuous), sex (male, female), race (non-Hispanic White, Black, Mexican American, Hispanic, and other ethnicity), education level (less than high school, high school, more than high school, or missing) and PIR (<1.3, 1.3–3.5, >3.5 or missing). Model 3 was adjusted for Model 2 plus hypertension (yes, no, or missing), diabetes (yes, no, or missing) and energy intake (continuous).

In addition, weighted restricted cubic spline analyses (RCS) with four knots were conducted to explore the dose–response relationship between OBS and the risk of FM% defined obesity as well as segmental body composition parameters. Age, gender, race, education level, family poverty income ratio, diabetes, hypertension and energy intake were adjusted as covariates. In the exploratory analyses, subgroup analyses by age, sex, race, PIR, education level and energy intake were performed. Sensitivity analyses by restricted the analysis to participants with the data of C-reactive protein (CRP) data were conducted. All analyses were performed using EmpowerStats 2.0 and R version 3.6.2; a *p*-value <0.05 with a two-sided test was considered to indicate statistical significance.

## 3 Results

### 3.1 Baseline characteristics

**Figure 1:**
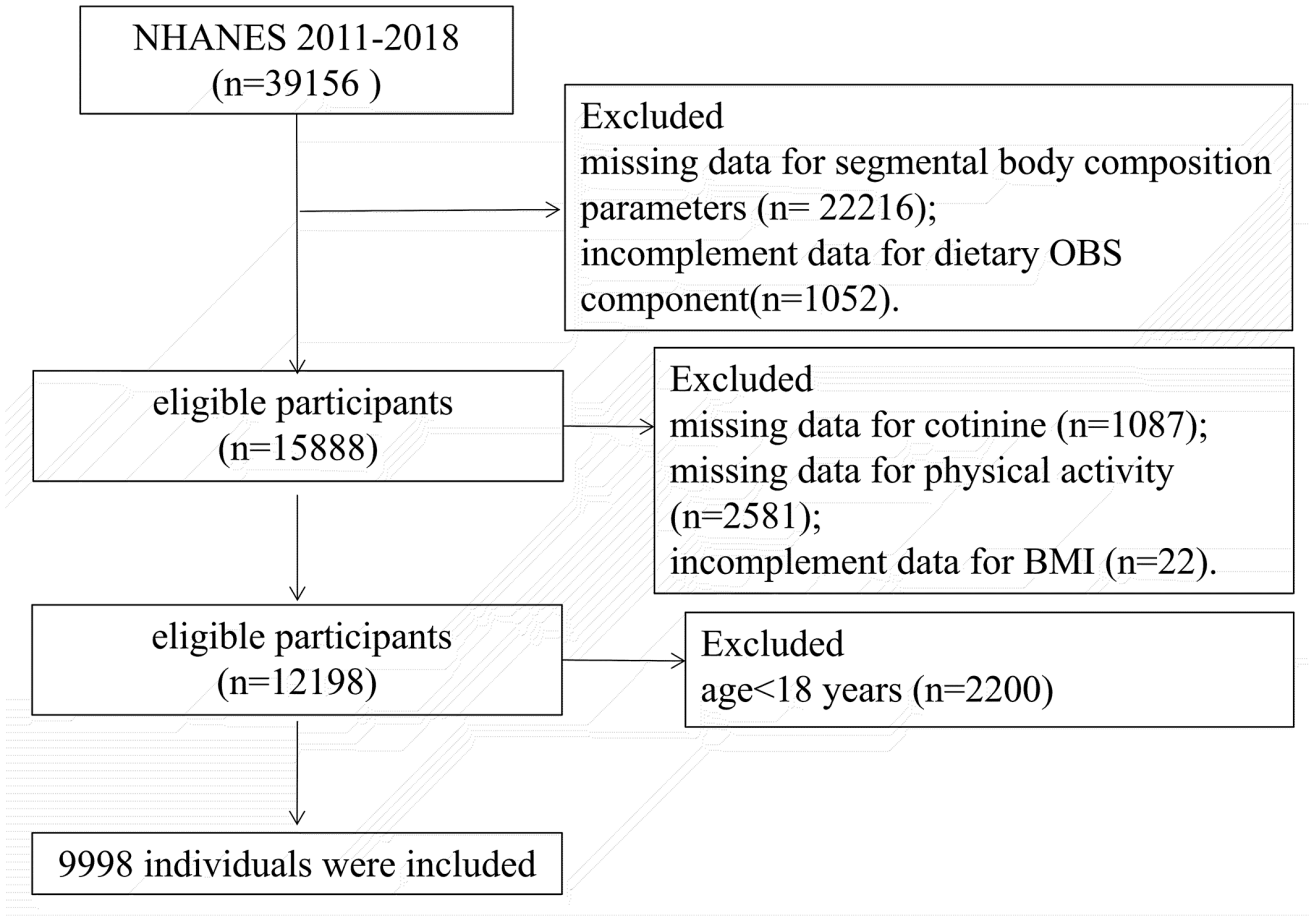
The flowchart of the sample selection.

Participants aged 18 to 59 years who completed the DXA examinations between 2011 and 2018 were included. We excluded pregnancy, individuals who weighed more than 450 pounds or taller than 6′5″ and other cases whose DXA examination were invalid. Individuals were also excluded for missing any data on the OBS components (Supplementary Table S1). Finally, 9,998 participants were enrolled. The flowchart is shown in Figure 1.

The baseline characteristics of the individuals based on quartiles of the OBS are shown in Table 1. Age, sex, race/ethnicity, family PIR, education level, hypertension status, energy intake, as well as segmental FM% and LM% were significantly different between the OBS quartiles (all *p* < 0.001). Participants in higher OBS quartiles were older and had a higher education level and incomes, but a lower energy intake. In addition, participants in higher OBS quartiles tended to have a lower incidence of FM%-defined obesity (Q1:74.20%, Q2: 69.78%, Q3: 66.60%, Q4: 62.20%, *p* trend <0.001) and BMI-defined obesity (Q1:43.47%, Q2: 36.72%, Q3: 34.50%, Q4: 25.39%, *p* trend <0.001) than those in the lowest quartile. Compared with those in Q1, participants in higher quartiles had higher segmental LM% while a lower FM% (all *p* < 0.001).

**Table 1:** The baseline characteristics of participants from National Health and Nutrition Examination Survey, United States, by quartiles of the OBS.

	Overall (2–47)	Q1 (2–18)	Q2 (19–24)	Q3 (25–30)	Q4 (31–47)	*p*-value
Age, year	38.13 ± 0.28	36.66 ± 0.44	38.01 ± 0.33	38.00 ± 0.35	39.45 ± 0.46	<0.001
Male, *n* (%)	5,068 (51.63)	1,092 (49.40)	1,301 (55.00)	1,299 (54.08)	1,376 (48.40)	<0.001
Race, *n* (%)						<0.001
Non-Hispanic White	3,466 (61.54)	879 (62.18)	869 (61.43)	812 (60.01)	906 (62.45)	
Non-Hispanic Black	2023 (10.67)	660 (16.13)	601 (12.79)	445 (9.53)	317 (5.81)	
Mexican American	1,585 (10.91)	279 (9.10)	390 (10.90)	447 (12.70)	469 (10.76)	
Other Hispanic	1,056 (7.44)	184 (5.68)	271 (8.20)	269 (7.48)	332 (8.08)	
Other race	1868 (9.44)	234 (6.91)	328 (6.68)	509 (10.28)	797 (12.90)	
Family PIR, *n* (%)						<0.001
<1.3	3,119 (23.99)	890 (31.15)	840 (26.57)	743 (23.33)	646 (17.10)	
1.3–3.5	3,277 (34.53)	749 (37.33)	855 (38.34)	815 (34.80)	858 (29.06)	
>3.5	2,827 (41.48)	443 (31.51)	573 (35.09)	724 (41.87)	1,087 (53.84)	
Education level, *n* (%)						<0.001
Less than high school	563 (3.68)	88 (2.87)	139 (3.91)	149 (3.70)	187 (4.07)	
High school	3,679 (32.52)	1,065 (45.54)	1,005 (36.30)	887 (31.09)	722 (20.94)	
More than high school	5,754 (63.80)	1,083 (51.59)	1,315 (59.80)	1,445 (65.21)	1911 (74.99)	
Diabetes, *n* (%)	667 (5.25)	136 (5.32)	155 (4.88)	188 (5.60)	188 (5.21)	0.835
Hypertension, *n* (%)	2,121 (20.70)	512 (21.32)	574 (23.42)	514 (20.87)	521 (17.83)	0.005
Energy intake, kcal/day	2,271.53 ± 13.14	2,459.73 ± 36.88	2,423.61 ± 26.59	2,288.93 ± 25.22	1,990.72 ± 18.91	<0.001
Arm FM%	33.24 ± 0.16	34.71 ± 0.35	32.88 ± 0.34	32.65 ± 0.29	32.96 ± 0.29	<0.001
Leg FM%	34.87 ± 0.16	36.17 ± 0.31	34.72 ± 0.29	34.23 ± 0.27	34.57 ± 0.26	<0.001
Torso FM%	31.48 ± 0.17	32.88 ± 0.31	31.50 ± 0.28	31.22 ± 0.27	30.63 ± 0.25	<0.001
Total FM%	32.51 ± 0.14	33.82 ± 0.28	32.44 ± 0.27	32.09 ± 0.24	31.95 ± 0.23	<0.001
Arm LM%	62.91 ± 0.15	61.55 ± 0.33	63.29 ± 0.32	63.52 ± 0.29	63.14 ± 0.28	<0.001
Leg LM%	61.80 ± 0.15	60.60 ± 0.30	61.95 ± 0.28	62.43 ± 0.26	62.04 ± 0.24	<0.001
Torso LM%	66.94 ± 0.16	65.59 ± 0.29	66.93 ± 0.27	67.20 ± 0.26	67.74 ± 0.24	<0.001
Total LM%	64.52 ± 0.14	63.29 ± 0.27	64.60 ± 0.25	64.93 ± 0.23	65.00 ± 0.22	<0.001
FM% defined obesity, *n* (%)	6,748 (67.74)	1,620 (74.20)	1,684 (69.78)	1,663 (66.60)	1781 (62.20)	<0.001
BMI defined obesity, *n* (%)	3,469 (34.32)	991 (43.47)	929 (36.72)	840 (34.50)	709 (25.39)	<0.001

Continuous variables are presented as means ± SE or numbers (percentages) for categorical variables unless otherwise indicated. *p* value was estimated using chi-square for proportions, variance analysis for means. All estimates accounted for complex survey designs, and all percentages were weighted. Family PIR, ratio of family income to poverty; FM%, fat mass percentage; LM%, lean mass percentage; OBS, oxidative balance scores.

### 3.2 Associations between OBS and the risk of obesity

The associations between OBS and the risk of obesity are shown in Table 2. Each 1-SD increase in the OBS was associated with a 29% lower OR of BMI-defined obesity (OR = 0.71, 95% CI = 0.66, 0.76; *p* < 0.001) in the fully adjusted model. Compared to those in the lowest quartile of OBS (Q1), participants in the highest quartile of OBS (Q4) had a 57% lower risk of BMI-defined obesity (OR = 0.43, 95% CI = 0.36, 0.50; *p* trend <0.001).

**Table 2:** Association between OBS and the risk of obesity.

Variable	OR (95% CI)		
BMI defined obesity^a^	Model 1	Model 2	Model 3
OBS, Per 1-SD increase	0.72 (0.68, 0.77)^*^	0.72 (0.67, 0.77)^*^	0.71 (0.66, 0.76)^*^
OBS categories			
Quartile 1	1.00 (Ref.)	1.00 (Ref.)	1.00 (Ref.)
Quartile 2	0.75 (0.65, 0.87)^*^	0.73 (0.63, 0.84)^*^	0.72 (0.62, 0.82)^*^
Quartile 3	0.69 (0.58, 0.81)^*^	0.67 (0.56, 0.81)^*^	0.66 (0.55, 0.80)^*^
Quartile 4	0.44 (0.38, 0.51)^*^	0.44 (0.37, 0.51)^*^	0.43 (0.36, 0.50)^*^
P for trend	<0.001	<0.001	<0.001
FM% defined obesity^b^	Model 1	Model 2	Model 3
OBS, Per 1-SD increase	0.80 (0.75, 0.85)^*^	0.75 (0.70, 0.80)^*^	0.71 (0.66, 0.76)^*^
OBS categories			
Quartile 1	1.00 (Ref.)	1.00 (Ref.)	1.00 (Ref.)
Quartile 2	0.80 (0.65, 1.00)	0.74 (0.60, 0.92)^*^	0.72 (0.58, 0.89)^*^
Quartile3	0.69 (0.57, 0.84)^*^	0.63 (0.51, 0.76)^*^	0.59 (0.48, 0.73)^*^
Quartile 4	0.57 (0.48, 0.68)^*^	0.48 (0.40, 0.58)^*^	0.43 (0.35, 0.52)^*^
P for trend	<0.001	<0.001	<0.001

Model 1: Without adjustment.

Model 2: Adjusted for age, gender, race, education level and family poverty income ratio.

Model 3: Further adjusted for diabetes, hypertension and energy intake.

Data are expressed as OR (95% CI). OBS, oxidative balance scores.

*p < 0.01.

a BMI (in kg/m^2^) ≥ 30 was defined as obesity according to clinical guidelines.

b An FM% ≥ 25% for men or an FM% ≥ 35% for women was defined as obesity.

According to the multivariate model, per 1-SD increase in the OBS was associated with a 29% lower OR of FM%-defined obesity (OR = 0.71, 95% CI = 0.66, 0.76; *p* < 0.001). Compared to those in the lowest quartile of OBS (Q1), participants in the highest quartile of OBS (Q4) had a 57% lower risk of FM%-defined obesity (OR = 0.43, 95% CI = 0.35, 0.52; *p* trend <0.001).

**Figure 2:**
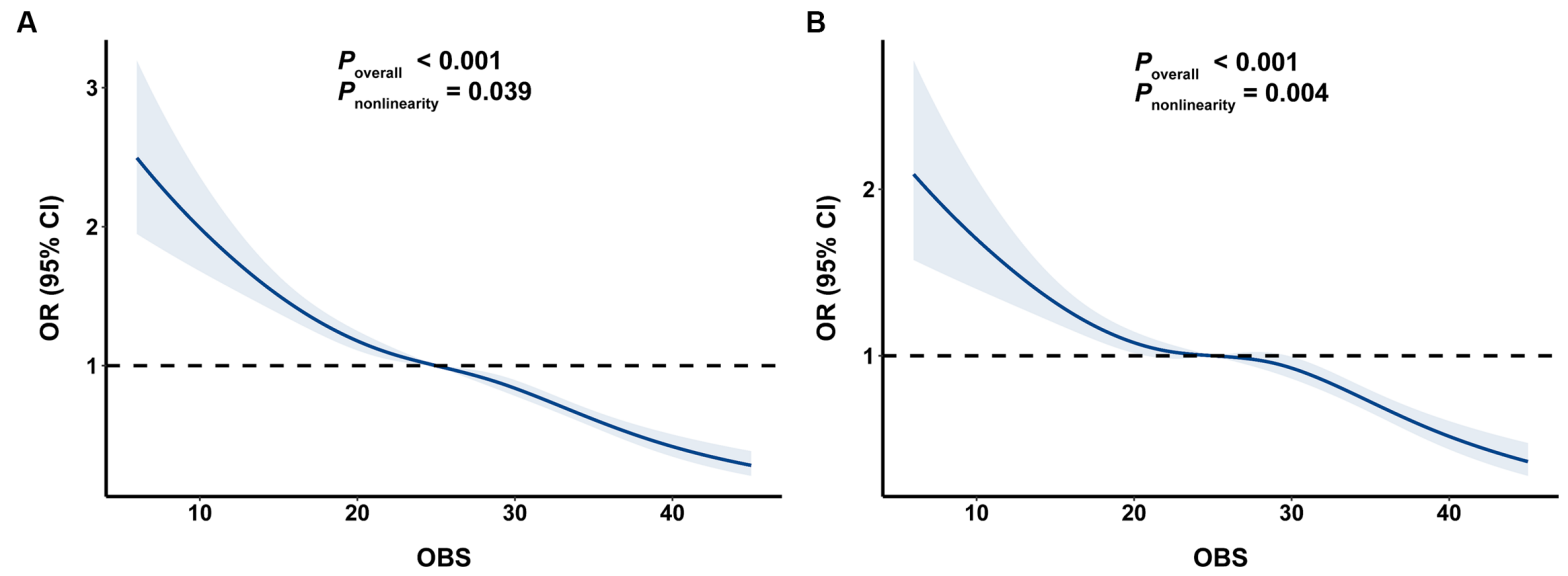
The associations of OBS with risk obesity. **(A)** OBS and BMI-defined obesity, **(B)** OBS and FM%-defined obesity. OBS, oxidative balance score; BMI, body mass index; FM%, fat mass percentage.

Additionally, as shown in Figure 2, RCS analysis revealed a significant negative relationship between OBS and the risk of BMI-defined obesity and FM% defined obesity (*p* overall <0.001).

### 3.3 Associations between OBS and segmental body composition parameters

**Figure 3:**
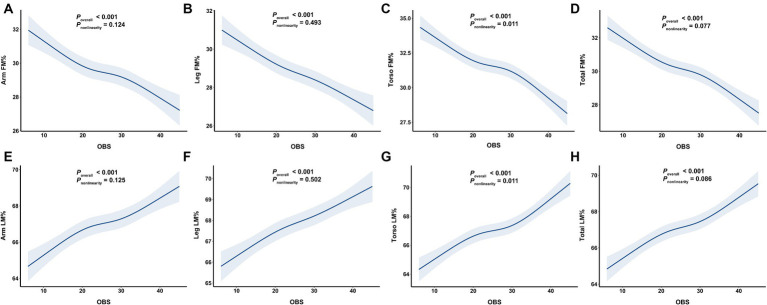
The associations of OBS with segmental body composition parameters. **(A)** OBS and arm FM%, **(B)** OBS and leg FM%, **(C)** OBS and torso FM%, **(D)** OBS and total FM%, **(E)** OBS and arm LM%, **(F)** OBS and leg LM%, **(G)** OBS and torso LM%, **(H)** OBS and total LM%. OBS, oxidative balance score; FM%, fat mass percentage; LM%, lean mass percentage.

Overall, we observed a significant negative association between OBS and segmental FM% and a positive between OBS and LM% (Figure 3). As shown in Table 3, the multivariable linear regression model showed that each 1-SD increase in OBS was negatively associated with segmental FM%, with β estimates (95% CIs) of −1.07 (−1.27, −0.86), −0.86 (1.04, −0.68), −1.32 (−1.52, −1.12), and − 1.06 (−1.23, −0.89) for arm FM%, leg FM%, torso FM% and total FM%, respectively. Similarly, a 1-SD increase in the OBS was positively associated with segmental LM%, with β estimates (95% CIs) of 0.99 (0.80, 1.18), 0.79 (0.62, 0.96), 1.26 (1.07, 1.45), and 0.98 (0.83, 1.14) for the arm LM%, leg LM%, torso LM% and total LM%, respectively. RCS analyses further revealed a negative association between OBS and segmental FM%, but a positive association between OBS and LM% (all *p* overall <0.001).

**Table 3:** The association between the OBS with segmental body composition parameters.

	OBS	Per 1-SD increase	Q1 (2–18)	Q2 (19–24)	Q3 (25–30)	Q4 (31–47)	*p* for trend
		β (95%CI)	β (95%CI)	β (95%CI)	β (95%CI)	β (95%CI)	
Arm FM%	Model 1	−0.60 (−0.88, −0.32)^*^	Ref	−1.81 (−2.75, −0.88)^*^	−2.05 (−3.03, −1.07)^*^	−1.74 (−2.56, −0.92)^*^	<0.001
	Model 2	−0.91 (−1.10, −0.72)^*^	Ref	−1.11 (−1.69, −0.53)^*^	−1.56 (−2.15, −0.97)^*^	−2.38 (−2.94, −1.81)^*^	<0.001
	Model 3	−1.07 (−1.27, −0.86)^*^	Ref	−1.18 (−1.74, −0.61)^*^	−1.75 (−2.35, −1.16)^*^	−2.76 (−3.35, −2.17)^*^	<0.001
Leg FM%	Model 1	−0.56 (−0.82, −0.31)^*^	Ref	−1.45 (−2.25, −0.65)^*^	−1.94 (−2.78, −1.10)^*^	−1.60 (−2.32, −0.88)^*^	<0.001
	Model 2	−0.72 (−0.90, −0.54)^*^	Ref	−0.69 (−1.22, −0.17)^*^	−1.32 (−1.85, −0.78)^*^	−1.85 (−2.37, −1.33)^*^	<0.001
	Model 3	−0.86 (−1.04, −0.68)^*^	Ref	−0.77 (−1.29, −0.24)^*^	−1.47 (−2.01, −0.94)^*^	−2.19 (−2.72, −1.65)^*^	<0.001
Torso FM%	Model 1	−0.85 (−1.07, −0.64)^*^	Ref	−1.38 (−2.19, −0.57)^*^	−1.67 (−2.49, −0.84)^*^	−2.25 (−2.91, −1.59)^*^	<0.001
	Model 2	−1.17 (−1.36, −0.97)^*^	Ref	−1.27 (−1.87, −0.66)^*^	−1.71 (−2.35, −1.06)^*^	−3.01 (−3.57, −2.45)^*^	<0.001
	Model 3	−1.32 (−1.52, −1.12)^*^	Ref	−1.34 (−1.94, −0.74)^*^	−1.89 (−2.53, −1.26)^*^	−3.37 (−3.94, −2.80)^*^	<0.001
Total FM%	Model 1	−0.69 (−0.90, −0.48)^*^	Ref	−1.39 (−2.13, −0.65)^*^	−1.73 (−2.51, −0.96)^*^	−1.88 (−2.50, −1.25)^*^	<0.001
	Model 2	−0.92 (−1.09, −0.76)^*^	Ref	−0.99 (−1.49, −0.48)^*^	−1.47 (−2.01, −0.93)^*^	−2.39 (−2.87, −1.91)^*^	<0.001
	Model 3	−1.06 (−1.23, −0.89)^*^	Ref	−1.06 (−1.56, −0.56)^*^	−1.64 (−2.17, −1.10)^*^	−2.72 (−3.22, −2.23)^*^	<0.001
Arm LM%	Model 1	0.54 (0.28, 0.81)^*^	Ref	1.73 (0.84, 2.62)^*^	1.96 (1.03, 2.90)^*^	1.58 (0.80, 2.36)^*^	<0.001
	Model 2	0.84 (0.66, 1.01)^*^	Ref	1.02 (0.48, 1.56)^*^	1.46 (0.91, 2.01)^*^	2.19 (1.66, 2.71)^*^	<0.001
	Model 3	0.99 (0.80, 1.18)^*^	Ref	1.09 (0.56, 1.62)^*^	1.65 (1.09, 2.20)^*^	2.56 (2.01, 3.11)^*^	<0.001
Leg LM%	Model 1	0.51 (0.26, 0.75)^*^	Ref	1.35 (0.60, 2.10)^*^	1.83 (1.03, 2.63)^*^	1.44 (0.76, 2.12)^*^	<0.001
	Model 2	0.66 (0.49, 0.82)^*^	Ref	0.62 (0.13, 1.11)^*^	1.23 (0.73, 1.73)^*^	1.68 (1.19, 2.17)^*^	<0.001
	Model 3	0.79 (0.62, 0.96)^*^	Ref	0.69 (0.20, 1.18)^*^	1.38 (0.88, 1.88)^*^	2.01 (1.51, 2.51)^*^	<0.001
Torso LM%	Model 1	0.81 (0.60, 1.02)^*^	Ref	1.34 (0.56, 2.12)^*^	1.62 (0.81, 2.42)^*^	2.15 (1.52, 2.79)^*^	<0.001
	Model 2	1.11 (0.93, 1.30)^*^	Ref	1.21 (0.62, 1.80)^*^	1.64 (1.01, 2.26)^*^	2.88 (2.34, 3.42)^*^	<0.001
	Model 3	1.26 (1.07, 1.45)^*^	Ref	1.28 (0.70, 1.87)^*^	1.82 (1.20, 2.43)^*^	3.23 (2.68, 3.78)^*^	<0.001
Total LM%	Model 1	0.62 (0.43, 0.82)^*^	Ref	1.31 (0.62, 2.01)^*^	1.64 (0.91, 2.38)^*^	1.71 (1.13, 2.30)^*^	<0.001
	Model 2	0.85 (0.70, 1.00)^*^	Ref	0.90 (0.43, 1.37)^*^	1.37 (0.87, 1.87)^*^	2.20 (1.75, 2.64)^*^	<0.001
	Model 3	0.98 (0.83, 1.14)^*^	Ref	0.97 (0.50, 1.43)^*^	1.53 (1.03, 2.03)^*^	2.52 (2.06, 2.98)^*^	<0.001

Model 1: Without adjustment.

Model 2: Adjusted for age, gender, race, education level and family income.

Model 3: Further adjusted for diabetes, hypertension and energy intake.

FM%, fat mass percentage; LM%, lean mass percentage; OBS, oxidative balance scores.

Data were expressed as β (95%CI).

*p < 0.05.

When OBS was treated as a categorical variable, higher OBS quartiles were associated with decreased segmental FM%; the β estimates (95% CIs) for the highest quartile (Q4) were − 2.76 (−3.35, −2.17), −2.19 (−2.72, −1.65), −3.37 (−3.94, −2.80), and − 2.72 (−3.22, −2.23) for the arm FM%, leg FM%, torso FM% and total FM%, respectively (all *p* trend <0.001), when compared with the lowest OBS quartile (Q1), suggesting a stable negative relationship between OBS and FM%. Accordingly, a higher OBS was associated with increased segmental LM%; the β estimates (95% CIs) for Q4 were 2.56 (2.01, 3.11), 2.01 (1.51, 2.51), 3.23 (2.68, 3.78) and 2.52 (2.06, 2.98) for the arm LM%, leg LM%, torso LM% and total LM%, respectively (reference to Q1) (all *p* trend <0.001).

### 3.4 Subgroup and sensitivity analyses

Subgroup analyses were also conducted to evaluate the possible effect modifications of the association between OBS and obesity as well as segmental FM% and LM%. After stratification by sex, age, race, Family PIR, education level and energy intake, the associations of OBS with obesity were inconsistent in different gender (both *p* interaction <0.01), race/ethnicity (both *p* interaction <0.05) and those with different education level (both *p* interaction <0.001), but remained consistent across categories of age, family PIR and energy intake (all *p* interaction >0.05) (Supplementary Tables S2, S3). Similar results were also observed for the associations between OBS and segmental FM% and LM%. Notably, the associations of OBS with Torso FM% and LM% remained consistent across the different age subgroups (*p* interaction >0.05), while the associations of OBS with limb FM% and LM% were stronger in those aged <40 years (*p* interaction <0.05) (Supplementary Tables S4–S9).

CRP is a reliable biomarker of inflammation (45). To adjust for possible confounding by CRP levels, we restricted the analysis to participants with the data of CRP data. Subjects with CRP levels had a similar OBS (25.62 ± 0.25) to those of people without CRP levels (25.42 ± 0.25, *p* = 0.576). Subjects with a higher OBS had a significantly lower CRP level (Supplementary Figure S2). After adjusting for age, sex, race, education level, family PIR, hypertension status, diabetes, energy intake and CRP level, the negative associations between OBS and obesity, as well as other segmental body composition parameters, were also stable (Supplementary Tables S10, S11).

## 4 Discussion

In the present nationwide cross-sectional study, we found that OBS was negatively associated with the risk of obesity and segmental FM% but positively associated with segmental LM%. These associations were independent of confounding factors and remained consistent in all the subgroup and sensitivity analyses. Accordingly, our results provide evidence for the importance of optimizing diet and lifestyle as crucial strategies in the prevention of segmental obesity in U.S. adults.

To our knowledge, this is the first study evaluating the association between OBS and FM% defined obesity as well as the percentage of segmental fat and lean mass. So far, previous epidemiological studies have explored OBS and the risk of central obesity. Noruzi et al. (32) reported that higher OBS was associated with a lower risk of abdominal obesity defined by high circumference, while another study suggested that there was no association between them in Tehranian adults (33). In addition, Yeo et al. (34) explored the association between OBS and central obesity and showed that Korean adults with a higher OBS had a smaller NC. Similar to our study, Wang et al. (36) reported that higher OBS was significantly correlated with lower risks of abdominal obesity and visceral fat accumulation. However, in Wang’s (36) study, they did not use segmental fat mass and lean mass, but focused on the association of OBS with total abdominal fat mass and visceral adipose tissue mass percentages. Our study reaffirms previous observations suggesting a dysregulated oxidative balance in individuals with obesity or unfavorable body composition. As for segmental body fat percentage, especially in the upper limbs and torso, may be more essential than overall when evaluating metabolic risk (12). Our results imply the significance of an anti-oxidative diet and lifestyle may be beneficial for metabolic disease. In addition, the identification of oxidative stress as a potential contributor to obesity underscores the importance of targeting oxidative balance in therapeutic interventions. Appropriate dietary and lifestyle modifications may hold promise in mitigating obesity-associated health risks and improving metabolic health outcomes in young and middle-aged adults.

Dietary status may directly impact our immune system and play a part in systemic chronic inflammation (46). Western diet, characterized by high consumption of red meat, refined grains and sugar-sweetened beverages can increase oxidative stress biomarkers (47). Within the health dietary models, the Mediterranean diet and the Dietary Approaches to Stop Hypertension (DASH) diet stand out. The Mediterranean diet (48), which emphasizes the intake of fruit and dairy, fish, poultry and wine, contributes to decrease in circulating oxidative stress biomarkers (49). The DASH diet, characterized by high consumption of fruits and vegetables, has the potential to reduce oxidative stress and inflammation levels (50). To some extent, combining various dietary and lifestyle factors to compose a comprehensive indicator could accurately indicate the physical oxidative stress level. OBS, in recent years, has attracted much attention due to its impact on health outcomes. OBS was found to be closely related to obesity (32), hypertension (51), chronic cardiovascular disease (45) and cancer (16). According to our results, OBS was negatively associated with the risk of obesity and segmental FM%. By elucidating the association between OBS and specific parameters of body composition, such as FM% and LM%, our findings offer mechanistic insights into the pathophysiology of obesity.

Although the association between OBS and obesity was not modified by age, PIR or energy intake (all *p* interaction >0.05). We observed significant sex, race and education level interactions, whereby the association of OBS with obesity were stronger in females, non-Hispanic White and Black individuals and people with more than high school education level. One reason for this gender discrepancy was that women have a greater antioxidant capacity than men, possibly owing to higher antioxidant enzyme activity (52) and higher free radicals scavenging ability in the presence of estrogen (53). Although the mechanism of this interaction remains to be elucidated, these findings may suggest that a predominance of antioxidant diet and lifestyle factors may be more protective against the gradual degeneration of lean mass in women, non-Hispanic White and Black and people with higher education levels.

Additionally, this study revealed that the associations of OBS with FM% and LM% of the trunk remained consistent in different age subgroups, while the associations of OBS with limb FM% and LM% were stronger in the younger group aged <40 years. Although the elderly individuals were prone to having an antioxidant dietary and life style, which had a higher OBS level (26.01 ± 0.24) than the middle-aged group (25.07 ± 0.18, *p* < 0.001), they still had a greater rate of obesity than did the younger individuals (76.44% vs. 60.09%, *p* < 0.001). One plausible mechanism explaining this disparity was that a higher OBS could not prevent the gradual degeneration of lean mass and the accumulation of fat mass with aging in elderly individuals (3, 4).

There are several strengths of this study. First, the present study with a large sample-size is based on data from the nationwide. Second, NHANES used a complex and multistage probability sampling design, and the present study adopted appropriate weighted analyses, so the findings is widely usable in the US population. Third, confounding factors, including sociodemographic characteristics and dietary intake are considered in weighted multiple regression analysis. Moreover, subgroup analyses confirm the results are basically robust. However, the limitations of this study cannot be neglected. First, because of the cross-sectional design and simultaneous measurement of exposure and results, it may be difficult to infer causality between OBS and segmental body composition. Therefore, prospective studies are required to further clarify the relationship. Second, the DXA data for participants aged ≥60 years old are not available, which limits the generalization to a wider age group (aged ≥60). Third, to date, no OBS biomarker is found to verify the effectiveness of OBS for assessing oxidative balance. Finally, although multiple potential confounding variables are adjusted in our analyses, residual confounding, such as medication use cannot be eliminated.

## 5 Conclusion

In conclusion, a higher OBS was negatively associated with the risk of FM%-defined obesity and positively associated with segmental lean mass. Our results underscore the significance of adhering to an anti-oxidative diet and lifestyle interventions for lowering segmental obesity in U.S. adults. However, further prospective studies are needed to verify our findings.

## Data availability statement

The datasets presented in this study can be found in online repositories. The names of the repository/repositories and accession number(s) can be found in the article/Supplementary material.

## Ethics statement

The studies involving humans were approved by the National Center for Health Statistics. The studies were conducted in accordance with the local legislation and institutional requirements. The participants provided their written informed consent to participate in this study.

## Author contributions

ZZ: Conceptualization, Formal analysis, Writing – original draft, Writing – review & editing. HB: Data curation, Formal analysis, Methodology, Validation, Writing – review & editing. ZL: Conceptualization, Data curation, Formal analysis, Methodology, Writing – original draft, Writing – review & editing. MF: Writing – original draft. GL: Validation, Writing – review & editing. LC: Writing – review & editing.
