# Supplementary material for: Association of the oxidative balance score with obesity and body composition among young and middle-aged adults
Source: Front Nutr. 2024 Apr 30;11:1373709. doi: 10.3389/fnut.2024.1373709 (PMC11095126; doi:10.3389/fnut.2024.1373709)
Supplement: Supplementary file 1 [file Data_Sheet_1.docx]

Association of the oxidative balance score with obesity and body composition among young and middle-aged adults

Supplementary Material

## Supplementary Figures


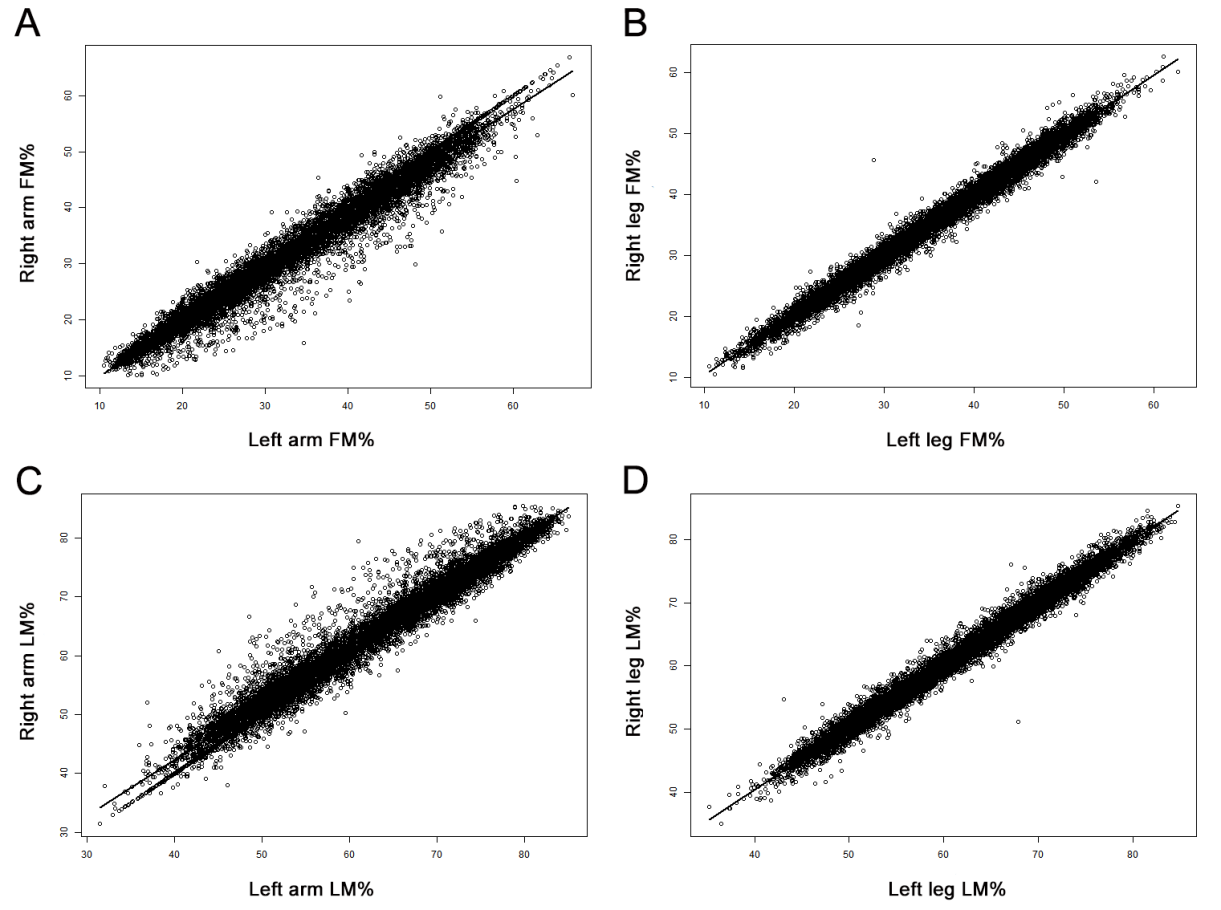


Supplementary Figure 1 The relationship between lean mass and body fat percentage in left and right. (A) FM% in the left arm and right arm; (B) FM% in the left leg and right leg; (C) LM% in the left arm and right arm; (D) LM% in the left leg and right leg. FM%, fat mass percentage; LM%, lean mass percentage.


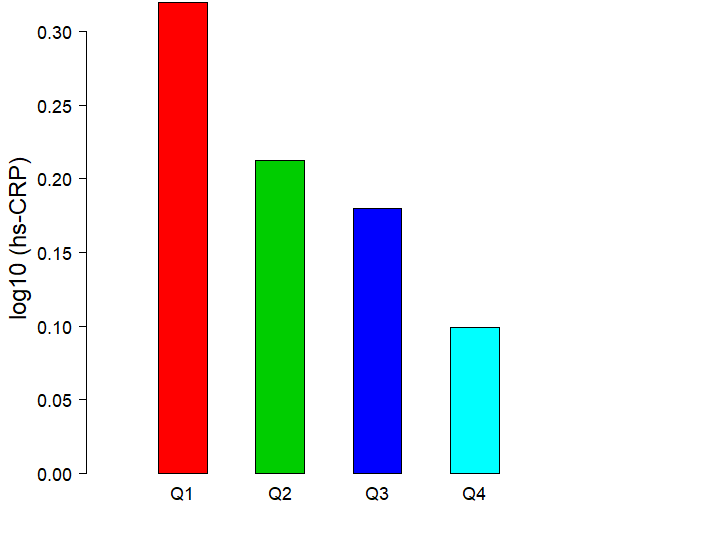


Supplementary Figure 2 CRP level in different quartiles of OBS

## Supplementary tables

Supplement Table S1 Oxidative balance score assignment scheme.

| OBS components | Property | Assignment scheme^a^ |
| --- | --- | --- |
| Dietary OBS components |  |  |
| 1. Dietary fiber (g/d) | A | 0 = Low (1st tertile), 1 = Medium (2nd tertile), 2 = High (3rd tertile) |
| 1. α-Carotene (mcg/d) | A | 0 = Low (1st tertile), 1 = Medium (2nd tertile), 2 = High (3rd tertile) |
| 1. β-Carotene (mcg/d) | A | 0 = Low (1st tertile), 1 = Medium (2nd tertile), 2 = High (3rd tertile) |
| 1. β-Cryptoxanthin (mcg/d) | A | 0 = Low (1st tertile), 1 = Medium (2nd tertile), 2 = High (3rd tertile) |
| 1. [Lycopene (mcg/d)](https://wwwn.cdc.gov/Nchs/Nhanes/2011-2012/DR1TOT_G.htm#DR1TLYCO) | A | 0 = Low (1st tertile), 1 = Medium (2nd tertile), 2 = High (3rd tertile) |
| 1. L[utein + zeaxanthin (mcg/d)](https://wwwn.cdc.gov/Nchs/Nhanes/2011-2012/DR1TOT_G.htm#DR1TLZ) | A | 0 = Low (1st tertile), 1 = Medium (2nd tertile), 2 = High (3rd tertile) |
| 1. Riboflavin (mg/d) | A | 0 = Low (1st tertile), 1 = Medium (2nd tertile), 2 = High (3rd tertile) |
| 1. Niacin (mg/d) | A | 0 = Low (1st tertile), 1 = Medium (2nd tertile), 2 = High (3rd tertile) |
| 1. Vitamin B6 (mg/d) | A | 0 = Low (1st tertile), 1 = Medium (2nd tertile), 2 = High (3rd tertile) |
| 1. Total folate (mg/d) | A | 0 = Low (1st tertile), 1 = Medium (2nd tertile), 2 = High (3rd tertile) |
| 1. Vitamin B12 (mcg/d) | A | 0 = Low (1st tertile), 1 = Medium (2nd tertile), 2 = High (3rd tertile) |
| 1. Vitamin C (mg/d) | A | 0 = Low (1st tertile), 1 = Medium (2nd tertile), 2 = High (3rd tertile) |
| 1. Vitamin E (mg/d) | A | 0 = Low (1st tertile), 1 = Medium (2nd tertile), 2 = High (3rd tertile) |
| 1. Calcium (mg/d) | A | 0 = Low (1st tertile), 1 = Medium (2nd tertile), 2 = High (3rd tertile) |
| 1. Magnesium (mg/d) | A | 0 = Low (1st tertile), 1 = Medium (2nd tertile), 2 = High (3rd tertile) |
| 1. Zinc (mg/d) | A | 0 = Low (1st tertile), 1 = Medium (2nd tertile), 2 = High (3rd tertile) |
| 1. Copper (mg/d) | A | 0 = Low (1st tertile), 1 = Medium (2nd tertile), 2 = High (3rd tertile) |
| 1. Selenium (mcg/d) | A | 0 = Low (1st tertile), 1 = Medium (2nd tertile), 2 = High (3rd tertile) |
| 1. [Vitamin D (D2 + D3) (mcg/d)](https://wwwn.cdc.gov/Nchs/Nhanes/2011-2012/DR1TOT_G.htm#DR1TVD) | A | 0 = Low (1st tertile), 1 = Medium (2nd tertile), 2 = High (3rd tertile) |
| 1. Total fat (g/d) | P | 0 = High (3rd tertile), 1 = Medium (2nd tertile), 2 = Low (1st tertile) |
| 1. Iron (mg/d) | P | 0 = High (3rd tertile), 1 = Medium (2nd tertile), 2 = Low (1st tertile) |
| Lifestyle OBS components |  |  |
| 1. Physical activity (MET/week) | A | 0 = Inactive group (no leisure-time physical activity), 1 = Insufficiently active group (leisure-time moderate activity 1–5 times per week or leisure-time vigorous activity 1–3 times per week), 2 = Active group (those who had more leisure-time moderate or vigorous activity than above) |
| 1. Alcohol (g/d) | P | 0 = heavy drinkers (≥30g/d for male and ≥15g/d for female), 1 = non-heavy drinkers (0 to 30g/d for male and 0 to 15g/d for female), 2 =Nondrinkers (0g/day) |
| 1. Body mass index (kg/m^2^) | P | 0 = High (3rd tertile), 1 = Medium (2nd tertile), 2 = Low (1st tertile) |
| 1. Cotinine (ng/mL) | P | 0 = High (3rd tertile), 1 = Medium (2nd tertile), 2 = Low (1st tertile) |

^a^ Low, medium, and high categories correspond to sex-specific 1st, 2nd, and 3rd tertiles.

OBS, oxidative balance scores.

Supplement Table S2 Subgroup analysis of association between OBS quartiles and the risk of obesity ^a^

|  | Per 1-SD increase | Q1 (2-18) | Q2 (19-24) | Q3 (25-30) | Q4 (31-47) | p for trend | p for interaction |
| --- | --- | --- | --- | --- | --- | --- | --- |
| Gender |  |  |  |  |  |  | 0.008 |
| Male | 0.74 (0.67, 0.81) | 1.00 (Ref.) | 0.76 (0.61, 0.95) | 0.70 (0.54, 0.92) | 0.48 (0.38, 0.59) | <0.001 |  |
| female | 0.69 (0.63, 0.76) | 1.00 (Ref.) | 0.68 (0.55, 0.82) | 0.63 (0.50, 0.78) | 0.38 (0.30, 0.49) | <0.001 |  |
| Age |  |  |  |  |  |  | 0.400 |
| age<40 | 0.68 (0.63, 0.74) | 1.00 (Ref.) | 0.72 (0.59, 0.88) | 0.59 (0.49, 0.71) | 0.38 (0.31, 0.48) | <0.001 |  |
| age≥40 | 0.75 (0.67, 0.85) | 1.00 (Ref.) | 0.73 (0.57, 0.94) | 0.77 (0.56, 1.06) | 0.50 (0.37, 0.67) | <0.001 |  |
| Race/ethnicity |  |  |  |  |  |  |  |
| Non-Hispanic White and Black | 0.70 (0.64, 0.77) | 1.00 (Ref.) | 0.73 (0.62, 0.87) | 0.66 (0.52, 0.82) | 0.42 (0.34, 0.53) | <0.001 | 0.034 |
| Other race | 0.70 (0.64, 0.75) | 1.00 (Ref.) | 0.68 (0.54, 0.84) | 0.63 (0.50, 0.81) | 0.39 (0.31, 0.49) | <0.001 |  |
| Family PIR |  |  |  |  |  |  | 0.308 |
| ≤3.5 | 0.73 (0.68, 0.79) | 1.00 (Ref.) | 0.71 (0.61, 0.82) | 0.68 (0.56, 0.82) | 0.44 (0.35, 0.55) | <0.001 |  |
| >3.5 | 0.69 (0.61,0.78) | 1.00 (Ref.) | 0.77 (0.55, 1.07) | 0.68 (0.49, 0.95) | 0.43 (0.32, 0.58) | <0.001 |  |
| Education level |  |  |  |  |  |  | <0.001 |
| High school and below | 0.79 (0.73, 0.85) | 1.00 (Ref.) | 0.68 (0.55, 0.84) | 0.70 (0.55, 0.90) | 0.57 (0.45, 0.72) | <0.001 |  |
| More than high school | 0.67 (0.61, 0.74) | 1.00 (Ref.) | 0.73 (0.57, 0.93) | 0.62 (0.50, 0.77) | 0.37 (0.29, 0.47) | <0.001 |  |
| Energy intake |  |  |  |  |  |  | 0.191 |
| Low (＜2062 kcal/day) | 0.73 (0.66, 0.80) | 1.00 (Ref.) | 0.71 (0.58, 0.87) | 0.64 (0.48, 0.86) | 0.44 (0.34, 0.57) | <0.001 |  |
| High (≥2062 kcal/day) | 0.70 (0.64, 0.77) | 1.00 (Ref.) | 0.72 (0.58, 0.88) | 0.68 (0.54, 0.87) | 0.42 (0.33, 0.52) | <0.001 |  |

multivariate logistic regression model was adjusted for age, gender, race, education level and family PIR, self-reported diabetes, self-reported hypertension and energy intake except for the stratified variables. Family PIR, ratio of family income to poverty; OBS, oxidative balance scores. Data are expressed as OR (95% CI). *p < 0.05

^a^ BMI (in kg/m^2^) ≥30 was defined as obesity according to clinical guidelines.

Supplement Table S3 Subgroup analysis of association between OBS quartiles and the risk of FM% defined obesity ^a^

|  | Per 1-SD increase | Q1 (2-18) | Q2 (19-24) | Q3 (25-30) | Q4 (31-47) | p for trend | p for interaction |
| --- | --- | --- | --- | --- | --- | --- | --- |
| Gender |  |  |  |  |  |  | < 0.001 |
| Male | 0.74 (0.68, 0.81)^*^ | 1.00 (Ref.) | 0.81 (0.61, 1.09) | 0.66 (0.50, 0.88)^*^ | 0.50 (0.38, 0.64)^*^ | <0.001 |  |
| female | 0.68 (0.62, 0.75)^*^ | 1.00 (Ref.) | 0.62 (0.47, 0.82)^*^ | 0.53 (0.41, 0.69)^*^ | 0.36 (0.27, 0.47)^*^ | <0.001 |  |
| Age |  |  |  |  |  |  | 0.162 |
| age<40 | 0.67 (0.62, 0.73)^*^ | 1.00 (Ref.) | 0.70 (0.55, 0.89)^*^ | 0.55 (0.44, 0.70)^*^ | 0.37 (0.30, 0.46)^*^ | <0.001 |  |
| age≥40 | 0.79 (0.71, 0.89)^*^ | 1.00 (Ref.) | 0.81 (0.56, 1.16) | 0.70 (0.49, 1.00) | 0.59 (0.42, 0.83)^*^ | 0.003 |  |
| Race/ethnicity |  |  |  |  |  |  | 0.035 |
| Non-Hispanic White and Black | 0.70 (0.64, 0.76)^*^ | 1.00 (Ref.) | 0.76 (0.59, 0.96)^*^ | 0.58 (0.46, 0.75)^*^ | 0.41(0.32, 0.52)^*^ | <0.001 |  |
| Other race | 0.76 (0.69, 0.84)^*^ | 1.00 (Ref.) | 0.66 (0.49, 0.90)^*^ | 0.65 (0.49, 0.87)^*^ | 0.49 (0.36, 0.66)^*^ | <0.001 |  |
| Family PIR |  |  |  |  |  |  | 0.185 |
| ≤3.5 | 0.78 (0.73, 0.84)^*^ | 1.00 (Ref.) | 0.72 (0.57, 0.85)* | 0.71(0.57, 0.88)^*^ | 0.50 (0.41, 0.61)^*^ | <0.001 |  |
| >3.5 | 0.62 (0.55, 0.69)^*^ | 1.00 (Ref.) | 0.75 (0.51, 1.12)^*^ | 0.48 (0.31, 0.72)^*^ | 0.33 (0.22, 0.49)^*^ | <0.001 |  |
| Education level |  |  |  |  |  |  | <0.001 |
| High school and below | 0.88 (0.79, 0.97)^*^ | 1.00 (Ref.) | 0.69 (0.54, 0.89)^*^ | 0.76 (0.57,1.00) | 0.69 (0.54, 0.89)^*^ | 0.015 |  |
| More than high school | 0.64 (0.58, 0.69)^*^ | 1.00 (Ref.) | 0.71 (0.53, 0.94)^*^ | 0.48 (0.38, 0.61)^*^ | 0.33 (0.26, 0.43)^*^ | <0.001 |  |
| Energy intake |  |  |  |  |  |  | 0.991 |
| Low (＜2062 kcal/day) | 0.77 (0.70, 0.85)^*^ | 1.00 (Ref.) | 0.68 (0.51, 0.89)^*^ | 0.61 (0.44, 0.86)^*^ | 0.51 (0.38, 0.68)^*^ | <0.001 |  |
| High (≥2062 kcal/day) | 0.70 (0.64, 0.77)^*^ | 1.00 (Ref.) | 0.76 (0.58, 0.98)^*^ | 0.61 (0.46, 0.80)^*^ | 0.40 (0.31, 0.52)^*^ | <0.001 |  |

multivariate logistic regression model was adjusted for age, gender, race, education level and family PIR, self-reported diabetes, self-reported hypertension and energy intake except for the stratified variables. Family PIR, ratio of family income to poverty; OBS, oxidative balance scores. Data are expressed as OR (95% CI). *p < 0.05

^a^ An FM% ≥ 25% for men or an FM% ≥ 35% for women was defined as obesity.

Supplement Table S4 The association between the OBS with segmental body composition parameters in different gender

|  | OBS | Per 1-SD increase | Q1 (2-18) | Q2 (19-24) | Q3 (25-30) | Q4 (31-47) | P for trend | p for interaction |
| --- | --- | --- | --- | --- | --- | --- | --- | --- |
| Arm FM% | Male | -0.86 (-1.10, -0.61)* | Ref | -0.61 (-1.33, 0.12) | -1.49 (-2.33, -0.65)* | -2.00 (-2.68, -1.32)* | ＜0.001 | <0.001 |
|  | Female | -1.25 (-1.55, -0.96)* | Ref | -1.76 (-2.70, -0.82)* | -1.95 (-2.70, -1.20)* | -3.47 (-4.34, -2.60)* | ＜0.001 |  |
| Leg FM% | Male | -0.79 (-1.04, -0.55)* | Ref | -0.65 (-1.41, 0.11) | -1.60 (-2.38, -0.83)* | -1.88 (-2.58, -1.17)* | ＜0.001 | 0.009 |
|  | Female | -0.91 (-1.13, -0.69)* | Ref | -0.88 (-1.59, -0.18)* | -1.23 (-1.81, -0.65)* | -2.46 (-3.16, -1.77)* | ＜0.001 |  |
| Torso FM% | Male | -1.00 (-1.25, -0.76)* | Ref | -0.58 (-1.36, 0.20) | -1.36 (-2.20, -0.52)* | -2.37 (-3.04, -1.71)* | ＜0.001 | <0.001 |
|  | Female | -1.56 (-1.86, -1.27)* | Ref | -2.07 (-2.85, -1.29)* | -2.29 (-3.10, -1.49)* | -4.22 (-5.04, -3.41)* | ＜0.001 |  |
| Total FM% | Male | -0.86 (-1.08, -0.64)* | Ref | -0.58 (-1.26, 0.11) | -1.39 (-2.13, -0.64)* | -2.04 (-2.65, -1.44)* | ＜0.001 | <0.001 |
|  | Female | -1.22 (-1.46, -0.98)* | Ref | -1.51 (-2.21, -0.82)* | -1.77 (-2.40, -1.14)* | -3.31 (-4.00, -2.63)* | ＜0.001 |  |
| Arm LM% | Male | 0.82 (0.59, 1.06)* | Ref | 0.60 (-0.09, 1.30) | 1.46 (0.68, 2.25)* | 1.94 (1.28, 2.59)* | ＜0.001 | <0.001 |
|  | Female | 1.14 (0.87, 1.42)* | Ref | 1.59 (0.70, 2.47)* | 1.77 (1.08, 2.46)* | 3.15 (2.33, 3.96)* | ＜0.001 |  |
| Leg LM% | Male | 0.75 (0.51, 0.98)* | Ref | 0.61 (-0.11, 1.33) | 1.56 (0.83, 2.29)* | 1.76 (1.09, 2.43)* | ＜0.001 | 0.012 |
|  | Female | 0.83 (0.62, 1.03)* | Ref | 0.78 (0.12, 1.44)* | 1.10 (0.56, 1.63)* | 2.23 (1.59, 2.88)* | ＜0.001 |  |
| Torso LM% | Male | 0.96 (0.73, 1.20)* | Ref | 0.55 (-0.21, 1.32) | 1.32 (0.51, 2.14)* | 2.28 (1.63, 2.93)* | ＜0.001 | <0.001 |
|  | Female | 1.50 (1.21, 1.78)* | Ref | 1.97 (1.22, 2.73)* | 2.19 (1.41, 2.97)* | 4.05 (3.27, 4.83)* | ＜0.001 |  |
| Total LM% | Male | 0.81 (0.61,1.01)* | Ref | 0.54 (-0.11, 1.19) | 1.33 (0.63, 2.03)* | 1.92 (1.34, 2.49)* | ＜0.001 | <0.001 |
|  | Female | 1.12 (0.90, 1.34)* | Ref | 1.38 (0.73, 2.03)* | 1.62 (1.03, 2.20)* | 3.04 (2.40, 3.68)* | ＜0.001 |  |

Multivariate linear regression model was adjusted for age, race, education level and ratio of family income to poverty, diabetes, hypertension and energy intake. FM%, fat mass percentage; LM%, lean mass percentage; OBS, oxidative balance scores. Data were expressed as β (95%CI). *p < 0.05

Supplement Table S5 The association between the OBS with segmental body composition parameters in different age

|  | OBS | Per 1-SD increase | Q1 (2-18) | Q2 (19-24) | Q3 (25-30) | Q4 (31-47) | p for trend | p for interaction |
| --- | --- | --- | --- | --- | --- | --- | --- | --- |
| Arm FM% | age<40 | -1.31 (-1.59, -1.03)* | Ref | -0.99 (-1.67, -0.31)* | -2.15 (-2.89, -1.42)* | -3.23 (-4.06, -2.40)* | ＜0.001 | 0.040 |
|  | age≥40 | -0.71 (-1.01, -0.41)* | Ref | -1.26 (-2.29, -0.23)* | -1.14 (-2.02, -0.27)* | -1.99 (-2.86, -1.13)* | ＜0.001 |  |
| Leg FM% | age<40 | -1.18 (-1.45, -0.92)* | Ref | -0.76 (-1.35, -0.18 )* | -1.96 (-2.66, -1.27)* | -2.92 (-3.67, -2.18)* | ＜0.001 | 0.027 |
|  | age≥40 | -0.44 (-0.68, -0.19)* | Ref | -0.67 (-1.64, 0.29) | -0.77 (-1.58, 0.03) | -1.20 (-2.00, -0.40)* | 0.003 |  |
| Torso FM% | age<40 | -1.54 (-1.81, -1.26)* | Ref | -1.17 (-1.90, -0.44)* | -2.34 (-3.11, -1.57)* | -3.77 (-4.54, -2.99)* | ＜0.001 | 0.058 |
|  | age≥40 | -0.95 (-1.19, -0.70)* | Ref | -1.32 (-2.22, -0.41)* | -1.17 (-2.06, -0.28)* | -2.52 (-3.24, -1.81)* | ＜0.001 |  |
| Total FM% | age<40 | -1.31 (-1.55, -1.06)* | Ref | -0.96 (-1.56, -0.36)* | -2.08 (-2.74, -1.41)* | -3.21 (-3.91, -2.52)* | ＜0.001 | 0.034 |
|  | age≥40 | -0.70 (-0.92, -0.48)* | Ref | -1.01 (-1.86, -0.17)* | -0.96 (-1.73, -0.20)* | -1.89 (-2.56, -1.23)* | ＜0.001 |  |
| Arm LM% | age<40 | 1.22 (0.96, 1.49)* | Ref | 0.90 (0.27, 1.53)* | 2.01 (1.33, 2.70)* | 3.01 (2.23, 3.79)* | ＜0.001 | 0.038 |
|  | age≥40 | 0.66 (0.37, 0.94)* | Ref | 1.18 (0.20, 2.16)* | 1.09 (0.27, 1.91)* | 1.84 (1.03, 2.65)* | ＜0.001 |  |
| Leg LM% | age<40 | 1.10 (0.85, 1.35)* | Ref | 0.68 (0.14, 1.23)* | 1.83 (1.18, 2.48)* | 2.71 (2.01, 3.41)* | ＜0.001 | 0.026 |
|  | age≥40 | 0.39 (0.16, 0.62)* | Ref | 0.60 (-0.30, 1.51) | 0.73 (-0.03, 1.49) | 1.07 (0.33, 1.82)* | 0.004 |  |
| Torso LM% | age<40 | 1.47 (1.21, 1.73)* | Ref | 1.12 (0.42, 1.82)* | 2.25 (1.51, 2.99)* | 3.60 (2.86, 4.34)* | ＜0.001 | 0.058 |
|  | age≥40 | 0.91 (0.68, 1.15)* | Ref | 1.27 (0.39, 2.15)* | 1.13 (0.26, 1.99)* | 2.44 (1.76, 3.13)* | ＜0.001 |  |
| Total LM% | age<40 | 1.21 (0.98, 1.44)* | Ref | 0.87 (0.31, 1.42)* | 1.94 (1.32, 2.56)* | 2.98 (2.33, 3.63)* | ＜0.001 | 0.026 |
|  | age≥40 | 0.64 (0.45, 0.84)* | Ref | 0.94 (0.15, 1.74)* | 0.90 (0.19, 1.62)* | 1.75 (1.14, 2.37)* | ＜0.001 |  |

Multivariate linear regression model was adjusted for gender, race, education level and ratio of family income to poverty, diabetes, hypertension and energy intake. FM%, fat mass percentage; LM%, lean mass percentage; OBS, oxidative balance scores.

Data were expressed as β (95%CI). *p < 0.05

Supplement Table S6 The association between the OBS with segmental body composition parameters in different race

|  | OBS | Per 1-SD increase | Q1 (2-18) | Q2 (19-24) | Q3 (25-30) | Q4 (31-47) | p for trend | p for interaction |
| --- | --- | --- | --- | --- | --- | --- | --- | --- |
| Arm FM% | Non-Hispanic white and black | -1.09 (-1.34, -0.84)^*^ | Ref | -1.10 (-1.75, -0.44)^*^ | -1.86 (-2.57, -1.14)^*^ | -2.82 (-3.57, -2.08)^*^ | ＜0.001 | 0.452 |
|  | Other race | -0.82 (-1.10, -0.54)^*^ | Ref | -1.15 (-1.98, -0.33)^*^ | -1.09 (-1.94, -0.24)^*^ | -2.17 (-3.01, -1.32)^*^ | ＜0.001 |  |
| Leg FM% | Non-Hispanic white and black | -0.87 (-1.09, -0.65)^*^ | Ref | -0.69 (-1.30, -0.08 )^*^ | -1.55 (-2.19, -0.91)^*^ | -2.21 (-2.87, -1.55)^*^ | ＜0.001 | 0.043 |
|  | Other race | -0.72 (-0.98, -0.46)^*^ | Ref | -0.78 (-1.58, 0.02) | -1.02 (-1.75, -0.29)^*^ | -1.87 (-2.64, -0.09)* | ＜0.001 |  |
| Torso FM% | Non-Hispanic white and black | -1.43 (-1.68, -1.17)^*^ | Ref | -1.35 (-1.99, -0.71)^*^ | -2.09 (-2.87, -1.31)^*^ | -3.66 (-4.39, -2.93)^*^ | ＜0.001 | 0.553 |
|  | Other race | -0.96 (-1.26, -0.67)^*^ | Ref | -1.10 (-1.95, -0.26)^*^ | -1.15 (-1.94, -0.36)^*^ | -2.51 (-3.35, -1.66)^*^ | ＜0.001 |  |
| Total FM% | Non-Hispanic white and black | -1.12 (-1.33, -0.90)^*^ | Ref | -1.02 (-1.56, -0.48)^*^ | -1.76 (-2.41, -1.11)^*^ | -2.87 (-3.50, -2.25)^*^ | ＜0.001 | 0.231 |
|  | Other race | -0.83 (-1.08, -0.58)^*^ | Ref | -0.95 (-1.69, -0.21)^*^ | -1.07 (-1.77, -0.37)^*^ | -2.16 (-2.89, -1.42)^*^ | ＜0.001 |  |
| Arm LM% | Non-Hispanic white and black | 1.01 (0.78, 1.25)^*^ | Ref | 1.01 (0.40, 1.63)^*^ | 1.73 (1.07, 2.40)^*^ | 2.62 (1.92, 3.32)^*^ | ＜0.001 | 0.506 |
|  | Other race | 0.75 (0.49, 1.02)* | Ref | 1.07 (0.30, 1.84)^*^ | 1.04 (0.26, 1.82)^*^ | 1.98 (1.19, 2.77)^*^ | ＜0.001 |  |
| Leg LM% | Non-Hispanic white and black | 0.80 (0.60, 1.01)^*^ | Ref | 0.62 (0.05, 1.20)^*^ | 1.45 (0.84, 2.05)^*^ | 2.04 (1.42, 2.65)^*^ | ＜0.001 | 0.061 |
|  | Other race | 0.65 (0.40, 0.89)^*^ | Ref | 0.68 (-0.06 1.43) | 0.94 (0.25, 1.62)^*^ | 1.66 (0.93, 2.39)^*^ | ＜0.001 |  |
| Torso LM% | Non-Hispanic white and black | 1.37 (1.13, 1.62)^*^ | Ref | 1.29 (0.67, 1.92)^*^ | 2.01 (1.26, 2.76)^*^ | 3.53 (2.82, 4.23)^*^ | ＜0.001 | 0.594 |
|  | Other race | 0.92 (0.63, 1.20)^*^ | Ref | 1.05 (0.24, 1.86)^*^ | 1.10 (0.33, 1.86)^*^ | 2.38 (1.56, 3.20)^*^ | ＜0.001 |  |
| Total LM% | Non-Hispanic white and black | 1.04 (0.84, 1.24)^*^ | Ref | 0.94 (0.43, 1.45)* | 1.65 (1.05, 2.25)* | 2.68 (2.10, 3.26)* | ＜0.001 | 0.305 |
|  | Other race | 0.75 (0.52, 0.99)^*^ | Ref | 0.87 (0.19, 1.56)* | 0.99 (0.34, 1.64)* | 1.95 (1.27, 2.64)* | ＜0.001 |  |

Multivariate linear regression model was adjusted for age, gender, education level and ratio of family income to poverty, diabetes, hypertension and energy intake. FM%, fat mass percentage; LM%, lean mass percentage; OBS, oxidative balance scores. Data were expressed as β(95%CI). *p < 0.05

Supplement Table S7 The association between the OBS with segmental body composition parameters in different income`

|  | OBS | Per 1-SD increase | Q1 (2-18) | Q2 (19-24) | Q3 (25-30) | Q4 (31-47) | p for trend | p for interaction |
| --- | --- | --- | --- | --- | --- | --- | --- | --- |
| Arm FM% | Family PIR≤3.5 | -0.86 (-1.11, -0.60)* | Ref | -1.21 (-1.91, -0.51)* | -1.47 (-2.16, -0.77)* | -2.27 (-3.03, -1.50)* | ＜0.001 | 0.346 |
|  | Family PIR＞3.5 | -1.30 (-1.66, -0.95)* | Ref | -1.18 (-2.21, -0.15)* | -1.99 (-3.10, -0.88)* | -3.26 (-4.37, -2.16)* | ＜0.001 |  |
| Leg FM% | Family PIR≤3.5 | -0.74 (-0.96, -0.51)* | Ref | -0.85 (-1.48, -0.23 )* | -1.31 (-1.89, -0.72)* | -1.95 (-2.63, -1.27)* | ＜0.001 | 0.775 |
|  | Family PIR＞3.5 | -0.99 (-1.28, -0.69)* | Ref | -0.73 (-1.65, 0.20) | -1.59 (-2.61, -0.57)* | -2.41 (-3.33, -1.49)* | ＜0.001 |  |
| Torso FM% | Family PIR≤3.5 | -1.05 (-1.28, -0.82)* | Ref | -1.20 (-1.90, -0.49)* | -1.64 (-2.34, -0.95)* | -2.80 (-3.48, -2.11)* | ＜0.001 | 0.059 |
|  | Family PIR＞3.5 | -1.62 (-1.94, -1.30)* | Ref | -1.81 (-2.81, -0.82)* | -2.14 (-3.25, -1.04)* | -4.08 (-5.14, -3.03)* | ＜0.001 |  |
| Total FM% | Family PIR≤3.5 | -0.86 (-1.07, -0.66)* | Ref | -1.01 (-1.60, -0.43)* | -1.42 (-2.01, -0.83)* | -2.30 (-2.92, -1.68)* | ＜0.001 | 0.189 |
|  | Family PIR＞3.5 | -1.28 (-1.55, -1.01)* | Ref | -1.27 (-2.12, -0.42)* | -1.82 (-2.78, -0.86)* | -3.21 (-4.10, -2.31)* | ＜0.001 |  |
| Arm LM% | Family PIR≤3.5 | 0.80 (0.56, 1.03)* | Ref | 1.11 (0.46, 1.77)* | 1.36 (0.70, 2.02)* | 2.10 (1.38, 2.82)* | ＜0.001 | 0.435 |
|  | Family PIR＞3.5 | 1.21 (0.88, 1.55)* | Ref | 1.10 (0.13, 2.07)* | 1.92 (0.89, 2.95)* | 3.04 (2.01, 4.08)* | ＜0.001 |  |
| Leg LM% | Family PIR≤3.5 | 0.68 (0.47, 0.89)* | Ref | 0.77 (0.17, 1.36)* | 1.21 (0.66, 1.76)* | 1.80 (1.17, 2.44)* | ＜0.001 | 0.847 |
|  | Family PIR＞3.5 | 0.90 (0.62, 1.18)* | Ref | 0.65 (-0.21, 1.52) | 1.51 (0.56, 2.47)* | 2.21 (1.34, 3.08)* | ＜0.001 |  |
| Torso LM% | Family PIR≤3.5 | 1.00 (0.78, 1.23)* | Ref | 1.14 (0.46, 1.82)* | 1.57 (0.90, 2.25)* | 2.67 (2.00, 3.33)* | ＜0.001 | 0.051 |
|  | Family PIR＞3.5 | 1.56 (1.25, 1.87)* | Ref | 1.75 (0.79, 2.71) | 2.07 (1.01, 3.13)* | 3.94 (2.92, 4.96)* | ＜0.001 |  |
| Total LM% | Family PIR≤3.5 | 0.80 (0.61, 0.99)* | Ref | 0.93 (0.38, 1.48) | 1.32 (0.76, 1.87)* | 2.12 (1.54, 2.70)* | ＜0.001 | 0.204 |
|  | Family PIR＞3.5 | 1.19 (0.94, 1.45)* | Ref | 1.17 (0.38, 1.96) | 1.73 (0.84, 2.61)* | 2.99 (2.15, 3.82)* | ＜0.001 |  |

Multivariate linear regression model was adjusted for age, gender, race, education level, diabetes, hypertension and energy intake. FM%, fat mass percentage; LM%, lean mass percentage；OBS, oxidative balance scores.

Data were expressed as β (95%CI). *p < 0.05

Supplement Table S8 The association between the OBS with segmental body composition parameters in different education level

|  | OBS | Per 1-SD increase | Q1 (2-18) | Q2 (19-24) | Q3 (25-30) | Q4 (31-47) | p for trend | p for interaction |
| --- | --- | --- | --- | --- | --- | --- | --- | --- |
| Arm FM% | High school and below | -0.62 (-0.90, -0.33)* | Ref | -1.18 (-1.85, -0.51)* | -1.24 (-2.04, -0.44)* | -1.34 (-2.06, -0.62)* | ＜0.001 | <0.001 |
|  | More than high school | -1.29 (-1.56, -1.02)* | Ref | -1.25 (-1.99, -0.52)* | -2.15 (-2.82, -1.48)* | -3.43 (-4.23, -2.63)* | ＜0.001 |  |
| Leg FM% | High school and below | -0.48 (-0.73, -0.23)* | Ref | -0.89 (-1.51, -0.28 )* | -1.15 (-1.85, -0.46)* | -0.95 (-1.57, -0.33)* | 0.003 | <0.001 |
|  | More than high school | -1.05 (-1.27, -0.82)* | Ref | -0.73 (-1.43, -0.03) * | -1.72 (-2.35, -1.08)* | -2.74 (-3.44, -2.04)* | ＜0.001 |  |
| Torso FM% | High school and below | -0.74 (-1.03, -0.45)* | Ref | -1.00 (-1.72, -0.28)* | -1.29 (-2.09, -0.49)* | -1.66 (-2.42, -0.90)* | ＜0.001 | <0.001 |
|  | More than high school | -1.62 (-1.86, -1.38)* | Ref | -1.72 (-2.46, -0.98)* | -2.44 (-3.13, -1.75)* | -4.28 (-5.01, -3.54)* | ＜0.001 |  |
| Total FM% | High school and below | -0.60 (-0.84, -0.36)* | Ref | -0.92 (-1.52, -0.33)* | -1.18 (-1.86, -0.49)* | -1.29 (-1.90, -0.69)* | ＜0.001 | <0.001 |
|  | More than high school | -1.30 (-1.51, -1.09)* | Ref | -1.25 (-1.88, -0.61)* | -2.03 (-2.62, -1.44)* | -3.44 (-4.09, -2.78)* | ＜0.001 |  |
| Arm LM% | High school and below | 0.58 (0.32, 0.84)* | Ref | 1.11 (0.49, 1.73)* | 1.18 (0.43, 1.92)* | 1.24 (0.58, 1.90)* | ＜0.001 | <0.001 |
|  | More than high school | 1.20 (0.95, 1.45)* | Ref | 1.14 (0.44, 1.84)* | 2.01 (1.38, 2.64)* | 3.19 (2.43, 3.94)* | ＜0.001 |  |
| Leg LM% | High school and below | 0.44 (0.21, 0.67)* | Ref | 0.81 (0.24, 1.38)* | 1.09 (0.44, 1.74)* | 0.85 (0.28, 1.43)* | 0.004 | 0.002 |
|  | More than high school | 0.97 (0.75, 1.18)* | Ref | 0.65 (0.00, 1.30)* | 1.60 (1.00, 2.20) | 2.52 (1.86, 3.18)* | ＜0.001 |  |
| Torso LM% | High school and below | 0.70 (0.42, 0.98)* | Ref | 0.96 (0.26, 1.65)* | 1.25 (0.48, 2.02)* | 1.57 (0.84, 2.30)* | ＜0.001 | <0.001 |
|  | More than high school | 1.56 (1.33, 1.78)* | Ref | 1.65 (0.93, 2.37)* | 2.34 (1.67, 3.01)* | 4.11 (3.40, 4.82)* | ＜0.001 |  |
| Total LM% | High school and below | 0.55 (0.33, 0.78)* | Ref | 0.86 (0.32, 1.41)* | 1.12 (0.48, 1.75)* | 1.18 (0.62, 1.73)* | ＜0.001 | <0.001 |
|  | More than high school | 1.21 (1.01, 1.41)* | Ref | 1.13 (0.54, 1.72)* | 1.88 (1.33, 2.43)* | 3.19 (2.57, 3.80)* | ＜0.001 |  |

Multivariate linear regression model was adjusted for age, gender, race, ratio of family income to poverty, diabetes, hypertension and energy intake. FM%, fat mass percentage; LM%, lean mass percentage；OBS, oxidative balance scores.

Data were expressed as β (95%CI). *p < 0.05

Supplement Table S9 The association between the OBS with segmental body composition parameters in different energy intake

|  | OBS | Per 1-SD increase | Q1 (2-18) | Q2 (19-24) | Q3 (25-30) | Q4 (31-47) | p for trend | p for interaction |
| --- | --- | --- | --- | --- | --- | --- | --- | --- |
| Arm FM% | Low(＜2062 kcal/day) | -0.88 (-1.16, -0.60)* | Ref | -1.72 (-2.68, -0.76)* | -1.93 (-2.82, -1.04)* | -2.56 (-3.41, -1.71)* | ＜0.001 | 0.749 |
|  | High (≥2062 kcal/day) | -1.06 (-1.34, -0.77)* | Ref | -0.78 (-1.43, -0.14)* | -1.46 (-2.29, -0.62)* | -2.64 (-3.44, -1.85)* | ＜0.001 |  |
| Leg FM% | Low(＜2062 kcal/day) | -0.65 (-0.88, -0.41)* | Ref | -1.02 (-1.79, -0.25 )* | -1.30 (-2.02, -0.58)* | -1.75 (-2.46, -1.04)* | ＜0.001 | 0.621 |
|  | High (≥2062 kcal/day) | -0.91 (-1.20, -0.62)* | Ref | -0.53 (-1.14, 0.08) | -1.41 (-2.17, -0.64)* | -2.29 (-3.09, -1.48)* | ＜0.001 |  |
| Torso FM% | Low(＜2062 kcal/day) | -1.14 (-1.40, -0.89)* | Ref | -1.83 (-2.61, -1.06)* | -1.98 (-2.77, -1.19)* | -3.18 (-3.93, -2.43)* | ＜0.001 | 0.596 |
|  | High (≥2062 kcal/day) | -1.26 (-1.56, -0.96)* | Ref | -0.98 (-1.70, -0.27)* | -1.66 (-2.59, -0.73)* | -3.14 (-3.95, -2.34)* | ＜0.001 |  |
| Total FM% | Low(＜2062 kcal/day) | -0.88 (-1.10, -0.65)* | Ref | -1.42 (-2.11, -0.73)* | -1.63 (-2.32, -0.93)* | -2.44 (-3.11, -1.77)* | ＜0.001 | 0.949 |
|  | High (≥2062 kcal/day) | -1.06 (-1.31, -0.80)* | Ref | -0.77 (-1.36, -0.19)* | -1.48 (-2.25, -0.70)* | -2.64 (-3.35, -1.94)* | ＜0.001 |  |
| Arm LM% | Low(＜2062 kcal/day) | 0.81 (0.55, 1.08)* | Ref | 1.61 (0.71, 2.51)* | 1.81 (0.97, 2.64)* | 2.37 (1.57, 3.16)* | ＜0.001 | 0.753 |
|  | High (≥2062 kcal/day) | 0.99 (0.72, 1.25)* | Ref | 0.71 (0.10, 1.32)* | 1.37 (0.60, 2.15)* | 2.46 (1.71, 3.21)* | ＜0.001 |  |
| Leg LM% | Low(＜2062 kcal/day) | 0.59 (0.36, 0.81)* | Ref | 0.93 (0.21, 1.66)* | 1.20 (0.51, 1.89)* | 1.60 (0.93, 2.27)* | ＜0.001 | 0.553 |
|  | High (≥2062 kcal/day) | 0.84 (0.57, 1.12)* | Ref | 0.46 (-0.11, 1.03) | 1.33 (0.61, 2.05)* | 2.11 (1.35, 2.86)* | ＜0.001 |  |
| Torso LM% | Low(＜2062 kcal/day) | 1.10 (0.85, 1.35)* | Ref | 1.76 (1.01, 2.51)* | 1.91 (1.15, 2.67)* | 3.06 (2.33, 3.79)* | ＜0.001 | 0.551 |
|  | High (≥2062 kcal/day) | 1.20 (0.92, 1.49)* | Ref | 0.93 (0.24, 1.62)* | 1.59 (0.70, 2.49)* | 3.00 (2.22, 3.77)* | ＜0.001 |  |
| Total LM% | Low(＜2062 kcal/day) | 0.81 (0.60, 1.02)* | Ref | 1.31 (0.66, 1.96)* | 1.50 (0.86, 2.15)* | 2.25 (1.63, 2.88)* | ＜0.001 | 0.957 |
|  | High (≥2062 kcal/day) | 0.98 (0.74, 1.22)* | Ref | 0.70 (0.16, 1.24)* | 1.39 (0.66, 2.11)* | 2.44 (1.78, 3.10)* | ＜0.001 |  |

Multivariate linear regression model was adjusted for age, gender, race, education level and ratio of family income to poverty, diabetes and hypertension. FM%, fat mass percentage; LM%, lean mass percentage；OBS, oxidative balance scores. Data were expressed as β (95%CI). *p < 0.05

Supplement Table S10 Association between OBS and the risk of obesity (n=4416)

| Variable | OR (95% CI) | | |
| --- | --- | --- | --- |
| BMI defined obesity ^a^ | Model 1 | Model 2 | Model 3 |
| OBS, Per 1-SD increase | 0.72 (0.66, 0.77) | 0.71 (0.65, 0.77) | 0.77 (0.71, 0.84) |
| OBS categories |  |  |  |
| Quartile 1 | 1.00 (Ref.) | 1.00 (Ref.) | 1.00 (Ref.) |
| Quartile 2 | 0.60 (0.50, 0.72) | 0.58 (0.49, 0.68) | 0.59 (0.49, 0.71) |
| Quartile3 | 0.63 (0.48, 0.82) | 0.61 (0.47, 0.79) | 0.68 (0.51, 0.90) |
| Quartile 4 | 0.42 (0.34, 0.50) | 0.40 (0.33, 0.50) | 0.52 (0.42, 0.63) |
| P for trend | < 0.001 | < 0.001 | < 0.001 |
| FM% defined obesity ^b^ | Model 1 | Model 2 | Model 3 |
| OBS, Per 1-SD increase | 0.77 (0.71, 0.84)^*^ | 0.72 (0.66, 0.78)^*^ | 0.73 (0.67, 0.80)^*^ |
| OBS categories |  |  |  |
| Quartile 1 | 1.00 (Ref.) | 1.00 (Ref.) | 1.00 (Ref.) |
| Quartile 2 | 0.81 (0.59, 1.11) | 0.76 (0.56, 1.04) | 0.79 (0.57, 1.10) |
| Quartile3 | 0.64 (0.47, 0.88)^*^ | 0.57 (0.42, 0.78)^*^ | 0.59 (0.42, 0.83)^*^ |
| Quartile 4 | 0.53 (0.43, 0.67)^*^ | 0.44 (0.35, 0.56)^*^ | 0.47 (0.36, 0.62)^*^ |
| P for trend | < 0.001 | < 0.001 | < 0.001 |

Model 1: Without adjustment.

Model 2: Adjusted for age, gender, race, education level and family poverty income ratio.

Model 3: Further adjusted for diabetes, hypertension, energy intake and log 10 transformed C-reactive protein.

Data are expressed as OR (95% CI). OBS, oxidative balance scores.

^*^p < 0.01.

a BMI (in kg/m^2^) ≥30 was defined as obesity according to clinical guidelines.

b An FM% ≥ 25% for men or an FM% ≥ 35% for women was defined as obesity.

Supplement Table S11 The association between the OBS with segmental body composition parameters.(n=4423）

|  | OBS | Per 1-SD increase | Q1 (2-18) | Q2 (19-24) | Q3 (25-30) | Q4 (31-47) | p for trend |
| --- | --- | --- | --- | --- | --- | --- | --- |
|  |  | β (95%CI) | β (95%CI) | β (95%CI) | β (95%CI) | β (95%CI) |  |
| Arm FM% | Model 1 | -0.65 (-1.17, -0.13)^*^ | Ref | -2.79 (-4.21, -1.36)^*^ | -2.67 (-4.47, -0.87)^*^ | -2.12 (-3.63, -0.60)^*^ | 0.041 |
|  | Model 2 | -1.00 (-1.30, -0.70)^*^ | Ref | -1.43 (-2.21, -0.64)^*^ | -1.72 (-2.78, -0.65)^*^ | -2.68 (-3.59, -1.78)^*^ | <0.001 |
|  | Model 3 | -0.68 (-0.90, -0.45)^*^ | Ref | -1.03 (-1.66, -0.40)^*^ | -1.13 (-1.95, -0.32)^*^ | -1.79 (-2.51, -1.06)^*^ | <0.001 |
| Leg FM% | Model 1 | -0.61 (-1.04, -0.18)^*^ | Ref | -1.76 (-3.04, -0.47)^*^ | -2.33 (-3.78, -0.88)^*^ | -1.77 (-2.97, -0.58)^*^ | 0.010 |
|  | Model 2 | -0.82 (-1.06, -0.58)^*^ | Ref | -0.43 (-1.19, 0.33) | -1.29 (-2.12, -0.46)^*^ | -2.00 (-2.74, -1.26)^*^ | <0.001 |
|  | Model 3 | -0.60 (-0.78, -0.42)^*^ | Ref | -0.19 (-0.79, 0.41) | -0.85 (-1.51, -0.20)^*^ | -1.39 (-2.00, -0.77)^*^ | <0.001 |
| Torso FM% | Model 1 | -0.96 (-1.31, -0.62)^*^ | Ref | -2.09 (-3.40, -0.78)^*^ | -2.11 (-3.60, -0.62)^*^ | -2.77 (-3.82, -1.72)^*^ | <0.001 |
|  | Model 2 | -1.31 (-1.59, -1.02)^*^ | Ref | -1.65 (-2.60, -0.69)^*^ | -1.97 (-3.10, -0.85)^*^ | -3.51 (-4.36, -2.67)^*^ | <0.001 |
|  | Model 3 | -0.92 (-1.14, -0.70)^*^ | Ref | -1.23 (-2.00, -0.45)^*^ | -1.32 (-2.18, -0.45)^*^ | -2.47 (-3.17, -1.78)^*^ | <0.001 |
| Total FM% | Model 1 | -0.77 (-1.12, -0.41)^*^ | Ref | -1.99 (-3.15, -0.81)^*^ | -2.17 (-3.56, -0.78)^*^ | -2.24 (-3.28, -1.21)^*^ | <0.001 |
|  | Model 2 | -1.04 (-1.27, -0.81)^*^ | Ref | -1.15 (-1.90, -0.40)^*^ | -1.62 (-2.55, -0.70)^*^ | -2.74 (-3.44, -2.04)^*^ | <0.001 |
|  | Model 3 | -0.73 (-0.90, -0.56)^*^ | Ref | -0.81 (-1.38, -0.24)^*^ | -1.08 (-1.77, -0.38)^*^ | -1.90 (-2.45, -1.35)^*^ | <0.001 |
| Arm LM% | Model 1 | 0.60 (0.10, 1.10)^*^ | Ref | 2.63 (1.28, 3.98)^*^ | 2.57 (0.84, 4.29)^*^ | 1.95 (0.50, 3.41)^*^ | 0.051 |
|  | Model 2 | 0.94 (0.65, 1.22)^*^ | Ref | 1.28 (0.56, 2.00)^*^ | 1.61 (0.62, 2.60)^*^ | 2.48 (1.64, 3.33)^*^ | <0.001 |
|  | Model 3 | 0.64 (0.43, 0.86)^*^ | Ref | 0.92 (0.34, 1.49)^*^ | 1.08 (0.32, 1.83)^*^ | 1.68 (1.02, 2.36)^*^ | <0.001 |
| Leg LM% | Model 1 | 0.55 (0.14, 0.97)^*^ | Ref | 1.59 (0.38, 2.81)^*^ | 2.19 (0.81, 3.57)^*^ | 1.60 (0.46, 2.73)^*^ | 0.014 |
|  | Model 2 | 0.75 (0.53, 0.98)^*^ | Ref | 0.31 (-0.40, 1.02) | 1.18 (0.40, 1.95)^*^ | 1.81 (1.11, 2.51)^*^ | <0.001 |
|  | Model 3 | 0.56 (0.39, 0.74)^*^ | Ref | 0.10 (-0.47, 0.66) | 0.79 (0.18, 1.40)^*^ | 1.27 (0.69, 1.86)^*^ | <0.001 |
| Torso LM% | Model 1 | 0.92 (0.59, 1.26)^*^ | Ref | 2.01 (0.75, 3.28)^*^ | 2.06 (0.61, 3.51)^*^ | 2.66 (164, 3.68)^*^ | <0.001 |
|  | Model 2 | 1.25 (0.98, 1.53)^*^ | Ref | 1.56 (0.64, 2.48)^*^ | 1.90 (0.81, 2.99)^*^ | 3.37 (2.56, 4.19)^*^ | <0.001 |
|  | Model 3 | 0.89 (0.67, 1.10)^*^ | Ref | 1.16 (0.41, 1.91)^*^ | 1.27 (0.43, 2.11)^*^ | 2.38 (1.71, 3.06)^*^ | <0.001 |
| Total LM% | Model 1 | 0.70 (0.36, 1.04)^*^ | Ref | 1.85 (0.74, 2.95)^*^ | 2.06 (0.74, 3.39)^*^ | 2.07 (1.08, 3.05)^*^ | <0.001 |
|  | Model 2 | 0.97 (0.75, 1.18)^*^ | Ref | 1.02 (0.32, 1.71)^*^ | 1.50 (0.64, 2.37)^*^ | 2.53 (1.88, 3.18)^*^ | <0.001 |
|  | Model 3 | 0.69 (0.53, 0.84)^*^ | Ref | 0.70 (0.17, 1.23)^*^ | 1.01 (0.36, 1.66)^*^ | 1.78 (1.26, 2.29)^*^ | <0.001 |

Model 1: Without adjustment.

Model 2: Adjusted for age, gender, race, education level and family income.

Model 3: Further adjusted for diabetes, hypertension, energy intake and log 10 transformed C-reactive protein.

FM%, fat mass percentage; LM%, lean mass percentage; OBS, oxidative balance scores.

Data were expressed as β (95%CI). ^*^p < 0.05
